# Histone Deacetylation Regulated by KDM1A to Suppress DACT1 in Proliferation and Migration of Cervical Cancer

**DOI:** 10.1155/2021/5555452

**Published:** 2021-07-24

**Authors:** Lingjuan Zeng, Chunyan Chen, Chanjiao Yao

**Affiliations:** No. 3 Obstetrics and Gynecology Department, Hunan Provincial People's Hospital, No. 61, West Jiefang Road, Furong District, Changsha, Hunan 410005, China

## Abstract

**Objective:**

Increased expression of KDM1A and decreased expression of DACT1 in cervical cancer cells were noticed in a previous study. This study is aimed at exploring the mechanism behind the KDM1A regulation on DACT1 in cervical cancer cells.

**Methods:**

The expression profile of KDM1A and DACT1 in cervical cancer tissues was searched in TCGA database. In vitro experiments verified the effect of KDM1A and DACT1 on proliferation and migration ability of cervical cancer cell lines after cell transfection. The interaction of KDM1A with HDAC1 was identified by coimmunoprecipitation (Co-IP). The expression levels of KDM1A and DACT1 in cervical cancer cell lines were determined by qRT-PCR and western blot.

**Results:**

TCGA database showed that cervical cancer tissues had elevated expression of KDM1A and decreased expression of DACT1, which was consistent with the observation in cervical cancer cell lines. KDM1A was found to negatively regulate DACT1 through histone deacetylation. Meanwhile, the downregulation of KDM1A or overexpression of DACT1 could suppress the cell proliferation and migration ability in HeLa and SiHa cells. Cotransfection of KDM1A and DACT1 overexpression could reverse the increased cell proliferation and migration ability induced by KDM1A overexpression.

**Conclusion:**

KDM1A can downregulate DACT1 expression through histone deacetylation and therefore suppress the proliferation and migration of cervical cancer cells.

## 1. Introduction

As the second most common malignant cancer in female, cervical cancer is characterized by poor prognosis in the advanced stage due to metastasis or recurrence [1]. Fortunately, this disease is curable in the early stage. Therefore, early screening as an effort to enhance early detection and treatment for cervical cancer is of paramount importance [2]. Human papilloma virus (HPV) is an infectious agent, and almost all cervical cancers are HPV-associated [3]. A total of 12 HPV genotypes are of high risk for cervical cancer, among which HPV 16 and HPV 18 are responsible for almost 70% of all infections [4]. Evidence in a Chinese cohort supported several DNA methylation markers as early detection biomarkers for cervical lesions [5]. Moreover, compounds with histone deacetylase (HDAC) inhibitory activity are proved to be attractive therapeutic approaches for cervical cancer considering the implication of epigenetic regulations in the tumorigenesis of this disease [6].

Acetylation refers to posttranscriptional modification, mainly including lysine acetylation, protein acetylation, and histone acetylation, among which histone acetylation is of critical importance for gene regulation [7]. Furthermore, two histone proteins, histone H3 acetyl K9 and histone H3 Tri Methyl K4, are closely associated with the overall survival of patients with cervical cancer [8]. The same literature also highlighted the importance of histone acetylation and deacetylation in the treatment of cervical cancer [7], but less information is available concerning how histone acetylation and deacetylation were regulated in cervical cancer and are therefore of high interest for a therapeutic use. The lysine-specific histone demethylase 1A (KDM1A/LSD1) is able to demethylate H3K4me1/2 and H3K9me1/2 and has emerged for its epigenetic regulation in carcinogenesis [9]. KDM1A is reported as an oncoprotein with upregulated expression detected in cancers, including hepatocellular carcinoma [10] and colorectal cancer [11]. But the implication of KDM1A in cervical cancer is not fully understood. Meanwhile, KDM1A was also reported to catalyze histone 3 demethylation and therefore to achieve gene repression [12]. In addition to that, KDM1A is generally found to have close association with HDAC [12]. Taking above information together, we hypothesized that KDM1A may also be implicated in cervical cancer development by regulation on certain gene expression through acetylation or deacetylation.

Typical searching in TCGA database identified that KDM1A was highly expressed in cervical cancer tissues. Further investigation in cervical cancer cell lines confirmed the highly expressed profile of KDM1A in cervical cell lines and also identified DACT1 as a target gene of KDM1A. In light of recent studies, overexpression of DACT1 was reported to suppress the proliferation, invasion, and migration ability of cervical cancer cells [13]. Therefore, we speculated that KDM1A may be able to downregulate DACT1 expression through histone deacetylation, to enhance the proliferation and migration of cervical cancer cells. Biological data in UCSC showed that DACT1 promoter can be influenced by histone 3 deacetylation. Therefore, this study was performed to verify whether KDM1A can regulate DACT1 expression to regulate biological functions of cervical cancer cells.

## 2. Materials and Method

### 2.1. Cell Culture

Human cervical cancer cell lines HeLa, Ca Ski, SiHa, C-33A, and C4-1 and immortalized human cervical epithelial cell line H8 were purchased from China Center for Type Culture Collection (CCTCC, Shanghai, China). All cells were maintained at DMEM (Gibco, NY, USA) with high glucose, in which 10% of fetal calf serum (FCS) and 1% mycillin were supplemented. The cells were cultured in a 37°C incubator with 5% CO_2_ and 95% humidity. Cells in logarithmic phase were harvested for following experiments.

### 2.2. Expression Profile of KDM1A and DACT1 in Human Cervical Cancer

The expression levels of TGFRB2 and DACT1 in human cervical cancer tissues were obtained from UALCAN (http://ualcan.path.uab.edu/) and GEPIA (http://gepia.cancer-pku.cn/detail.php?).

### 2.3. Cell Transfection

HeLa and SiHa cells in logarithmic phase were seeded in 6-well plates (2 × 10^5^/well) for cell culture of 24 h. After that, sh-KDM1A (2 *μ*g), pcDNA3.1-DACT1 (2 *μ*g), and corresponding negative controls (Genechem, Shanghai, China) were transfected into cells and named as the sh-KDM1A group, sh-NC group, pcDNA3.1-DACT1 group, and pcDNA3.1 group. Cells cotransfected with pcDNA3.1-KDM1A (2 *μ*g), pcDNA3.1-DACT1, (2 *μ*g) or negative controls were named as the pcDNA3.1 group, pcDNA3.1-KDM1A group, and pcDNA3.1-KDM1A+pcDNA3.1-DACT1 group. Lipfectamine 2000 transfection kit (Invitrogen, NY, USA) was used for cell transfection based on instructions on the kit.

### 2.4. Reverse Transcription Quantitative Polymerase Chain Reaction

TRIzol Reagent (Life Technologies, NY, USA) was used to extract the total RNA, whose purity and concentration were measured using a microplate reader (Biotek Synergy 2). The RNA was reversed into cDNA template on a PCR amplifier for real-time quantitative RT-PCR with the application of PCR analyzer (BIO-RAD, CFX Connect, USA). The expression of mRNA was relative to that of GAPDH. The PCR reaction was conducted at the condition of predenature of 95°C for 10 min, followed by 40 cycles of denature at 95°C for 10 s, annealing of 60°C for 20 s, and extension at 72°C for 34 s. Data were calculated using 2^-*ΔΔ*Ct^ [14]: ΔΔCt = [Ct_(target gene)_ − Ct_(internal gene)_]_experimental group_ − [Ct_(target gene)_ − Ct_(internal gene)_]_control group_. The primer sequences are listed in Table 1.

### 2.5. Western Blot

Cells were washed in precold PBS for 3 times before cell lysis for 30 min at ice with 100 *μ*L/50 mL cell lysate. Then, the cell lysis was centrifuged at 4°C and 12000 rpm for 10 min with supernatant collected. BCA kit (Vazyme, Nanjing, China) was used to detect the concentration of proteins. The proteins (loading volume of total cells 80 *μ*g, sediments of 30 *μ*L; the order was recorded) were separated on a 10% SDS-polyacrylamide gel and transferred into PVDF membrane (Millipore, Billerica, MA) to terminate the unspecific reaction with 5% skim milk powder for 1 h. After that, the membranes were incubated with rabbit anti human KDM1A (2139S, 1 : 1000, Cell Signaling Technology, Boston, USA), rabbit anti human DACT1 (ab42547, 1 : 1000, Abcam, Cambridge, USA), and rabbit anti human Anti-Histone H3 (acetyl K27, ab177178, 1 : 1000, Abcam, Cambridge, USA) for overnight at 4°C. The membranes were then washed with TBST for 3 × 10 min before incubation with horseradish peroxidase labeled goat anti rabbit IgG (1 : 5000, CoWin Biosciences, Beijing, China) at room temperature for 1 h. After TBST washing for 3 × 10 min, the membranes were analyzed using a chemiluminescence imaging system (Tannon, Shanghai, China). GAPDH was used as an internal control.

### 2.6. CCK8 Assay

CCK-8 kit (Dojindo Molecular Technologies, Inc., Japan) was used to assess the cell growth of HeLa and SiHa cells. After cell transfection, cells (5 × 10^3^/well) were seeded into 96-well plates for cell culture for 24 h, 48 h, 72 h, and 96 h, respectively, before incubation with CCK-8 solution for 2 h. The optical density was measured at the wavelength at 450 nm using a microplate reader (Biotek, USA).

### 2.7. Cell Clone Formation Assay

Cells in logarithmic phrase were digested with 0.25% trypsin and made into single cells. Cells floated in culture medium containing 10% FCS were seeded into a 37°C prewarmed culture dish (10 mL) at the density of 50, 100, and 200 cell per dish. The dishes were gently shaken to ensure cells were scattered evenly. Cells were cultured at 37°C and 5% humidity for 2~3 weeks. Cell culture was terminated when cell clones were visible by naked eyes. The supernatants in the culture dishes were abandoned, and cells were washed with PBS 2 times before fixation with 5 mL acetic acid/methanol (1 : 3) for 15 min. Abandon the fixation solution. Giemsa staining was performed for 10~30 min before cells were washed in running water and dried. The culture dishes were inverted to calculate the cell clones by the naked eyes or by a microscope with low power lens (10 or more cloned cells were counted). Cell clone formation rate was calculated after cell clone number was counted.

### 2.8. Wound Healing Assay

Cells in logarithmic phrase were seeded into 6-well plates (2 × 10^5^/well) for cell culture. Once cell confluence reaches 90%, cells were treated by mitomycin (1 *μ*g/mL) for 1 h, and a new 200 *μ*L pipette tip was used to make a straight scratch to the cells. Cells were washed with PBS twice and further cultured in high-glucose DMEM without serum. The cell migration ability was assessed and photographed under an inverted microscope at 0 h and 24 h, respectively: cell migration rate = ((0 h migration distance − 24 h migration distance)/0 h migration distance) × 100%.

### 2.9. Coimmunoprecipitation (Co-IP)

HeLa and SiHa cells in logarithmic phrase were added with precold cell lysis at 4°C for 15 min before centrifugation at 14000 g for 15 min. The supernatant was transferred into a new centrifuge tube for incubation with negative IgG for anti-KDM1A antibody (2139S, 1 : 50, Cell Signaling Technology, Boston, USA) or anti-H3 antibody (9649S, 1 : 25, Cell Signaling Technology, Boston, USA) for overnight at 4°C. The pretreated 10 *μ*L protein A Agarose beads were added into cell lysis for incubation at 4°C for 24 h, with gentle shaking to enable the coupling of antibody with protein A Agarose beads. Then, the cell lysis was centrifuged at 4°C and 3000 rpm for 3 min to allow the Agarose beads down to the tube bottom. The supernatants were removed, and the Agarose beads were washed in lysis buffer for 3 min before boiling water bath with 15 *μ*L 2 × SDS loading buffer. The expressions of DACT1 or Anti-Histone H3 (acetyl K27) in the protein complex were measured by western blot.

### 2.10. Statistical Analysis

GraphPad Prism 6.0 (GraphPad Software, Inc., La Jolla, CA) was used for data analysis. All data were calculated from averaged value of three repeat experiments and expressed as the mean ± standard deviation (SD). Comparison between two groups was analyzed using a *t*-test, and data among multiple groups were compared using one-way analysis of variance (ANOVA) and post hoc Tukey's tests. *p* < 0.05 was considered having statistically significance.

## 3. Results

### 3.1. Increased KDM1A Expression and Decreased DACT1 Expression in Cervical Cancer Tissues

**Figure 1:**
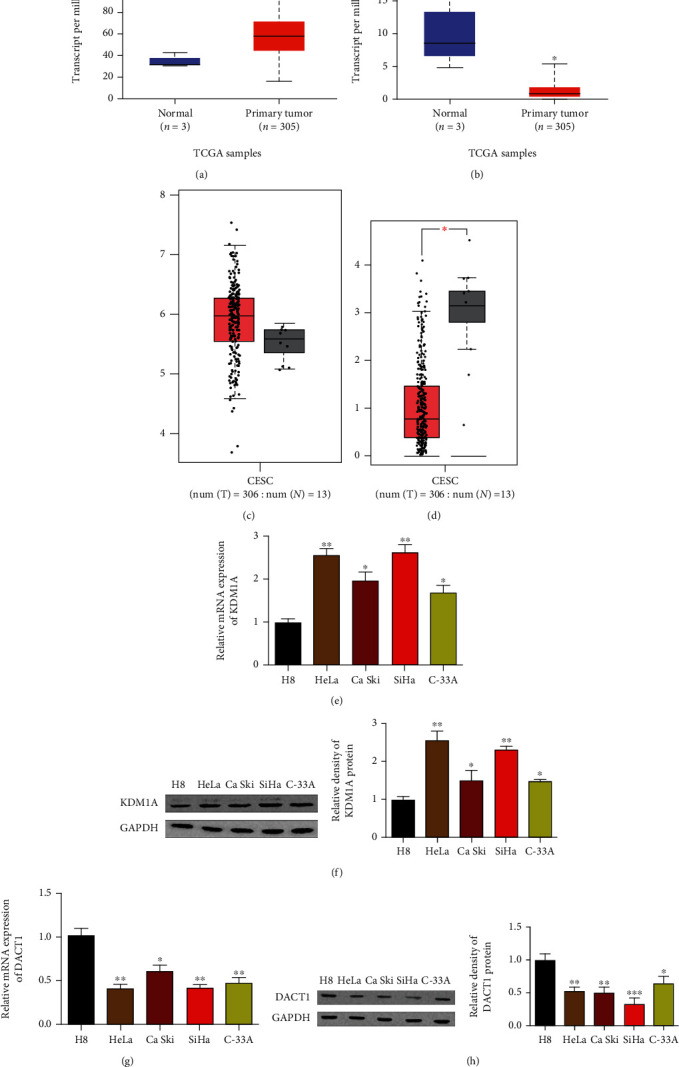
Measurement on expression profile of KDM1A and DACT1 in cervical cancer tissues showed highly expressed KDM1A and lowly expressed DACT1 in cervical cancer tissues. (a) KDM1A expression level in cervical cancer tissues from TCGA database in UALCAN (*n* = 305); (b) DACT1 expression level in cervical cancer tissues from TCGA database in UALCAN (*n* = 305); (c) KDM1A expression level in cervical cancer in GEPIA database (*n* = 306); (d) DACT1expression level in cervical cancer in GEPIA database (*n* = 306); (e) mRNA expression level of KDM1A in cervical cancer cell lines HeLa, Ca Ski, SiHa, and C-33A and immortalized human cervical epithelial cell line H8; (f) protein expression level of KDM1A in cervical cancer cell lines HeLa, Ca Ski, SiHa, and C-33A and immortalized human cervical epithelial cell line H8; (g) mRNA expression level of DACT1 in cervical cancer cell lines HeLa, Ca Ski, SiHa, and C-33A and immortalized human cervical epithelial cell line H8; (h) protein expression level of DACT1 in cervical cancer cell lines HeLa, Ca Ski, SiHa, and C-33A and immortalized human cervical epithelial cell line H8; ^∗^*p* < 0.05, ^∗∗^*p* < 0.01, and ^∗∗∗^*p* < 0.001, compared with H8 cells.

To fully understand the implication and effect of KDM1A and DACT1 in human cervical cancer tissues, we first searched the expression profile of those two factors in the UALCAN database (http://ualcan.path.uab.edu/). The results showed that KDM1A was highly expressed and DACT1 (305/3) was lowly expressed in cervical cancer tissues (Figures 1(a) and 1(b), *p* < 0.01). Similar expression pattern was also found in GEPIA database (http://gepia.cancer-pku.cn/detail.php?), in which increased KDM1A expression and deceased DACT1 expression (306/13) were found (Figures 1(c) and 1(d), *p* < 0.01).

Thereafter, we measured the mRNA and protein expression levels of KDM1A and DACT1 in cervical cancer cell lines (HeLa, Ca Ski, SiHa, and C-33A) and immortalized human cervical epithelial cell line H8. qRT-PCR and western blot demonstrated that compared with H8 cells, the mRNA (Figure 1(e), *p* < 0.01) and protein (Figure 1(f), *p* < 0.01) expressions of KDM1A were elevated, while those of DACT1 were decreased (Figures 1(g) and 1(h), *p* < 0.01) in all cervical cancer cell lines. Among the listed cervical cell lines, the maximum expression gap between KDM1A and DACT1 expression levels was found in HeLa and SiHa cells; therefore, HeLa and SiHa cells were selected for the following experiments. The above evidence suggested the implication of KDM1A and DACT1 in the progression of cervical cancer.

### 3.2. Knockdown of KDM1A Suppresses the Proliferation and Migration of Cervical Cancer Cells

**Figure 2:**
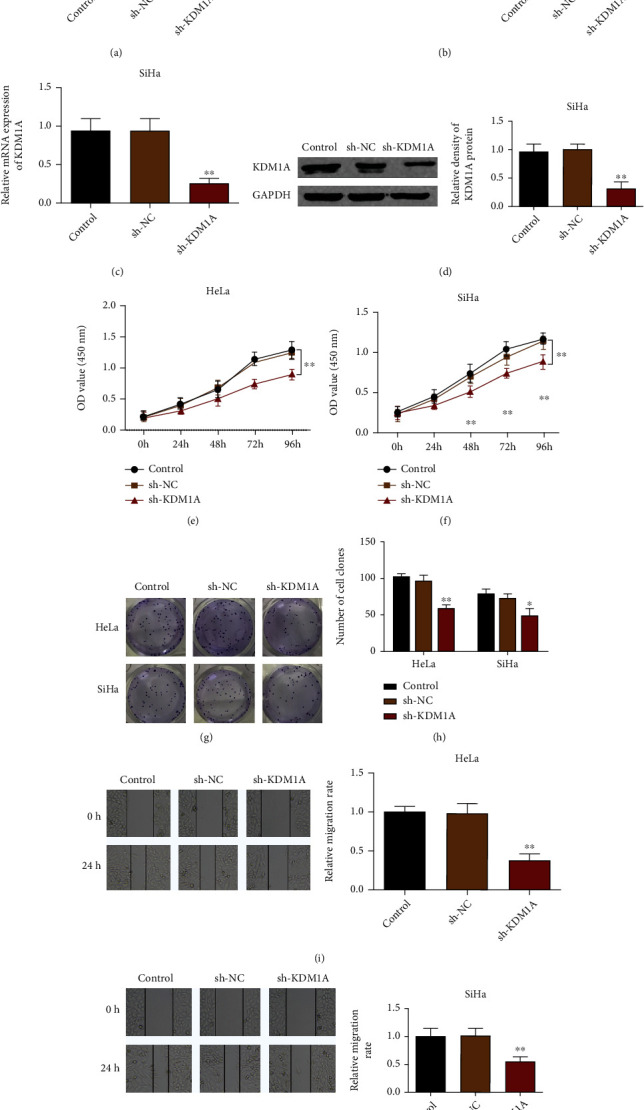
Knockdown of KDM1A contributes to the suppressed cell proliferation and migration of cervical cancer cells. (a) mRNA expression level of KDM1A in HeLa cells after KDM1A knockdown was determined by qRT-PCR; (b) protein expression level of KDM1A in HeLa cells after KDM1A knockdown was determined by western blot; (c) mRNA expression level of KDM1A in SiHa cells after KDM1A knockdown was determined by qRT-PCR; (d) protein expression level of KDM1A in SiHa cells after KDM1A knockdown was determined by western blot; (e) CCK8 assay was applied to assess the effect of KDM1A knockdown on proliferation of HeLa cells; (f) CCK8 assay was applied to assess the effect of KDM1A knockdown on proliferation of SiHa cells; (h, h) cell clone formation assay was used to assess the effect of KDM1A knockdown on cell clones in HeLa cells and SiHa cells; (i, j) wound healing assay analyzed the effect of KDM1A knockdown on cell migration in HeLa and SiHa cells. ^∗^*p* < 0.05 and ^∗∗^*p* < 0.01, compared with the control group.

To explore the effect of KDM1A on cervical cancer cells, we planned to transfect sh-KDM1A into HeLa and SiHa cells to achieve KDM1A suppression. The measurement on transfection efficiency showed that compared with the control group, HeLa and SiHa cells in the sh-KDM1A group had substantially decreased mRNA and protein expression levels of KDM1A (Figures 2(a)–2(d), *p* < 0.01). CCK8 and clone formation assay showed the sh-KDM1A group had suppressed proliferation rate (Figures 2(e) and 2(f)), decreased cell clones (Figures 2(g) and 2(h)), and inhibited migration rate (Figures 2(i) and 2(j)). No significant difference was found between the sh-NC group and control group. The above results showed that inhibition on KDM1A could suppress the proliferation and migration of HeLa and SiHa cells.

### 3.3. Overexpression of DACT1 Contributes to the Suppression on Proliferation and Migration of Cervical Cancer Cells

**Figure 3:**
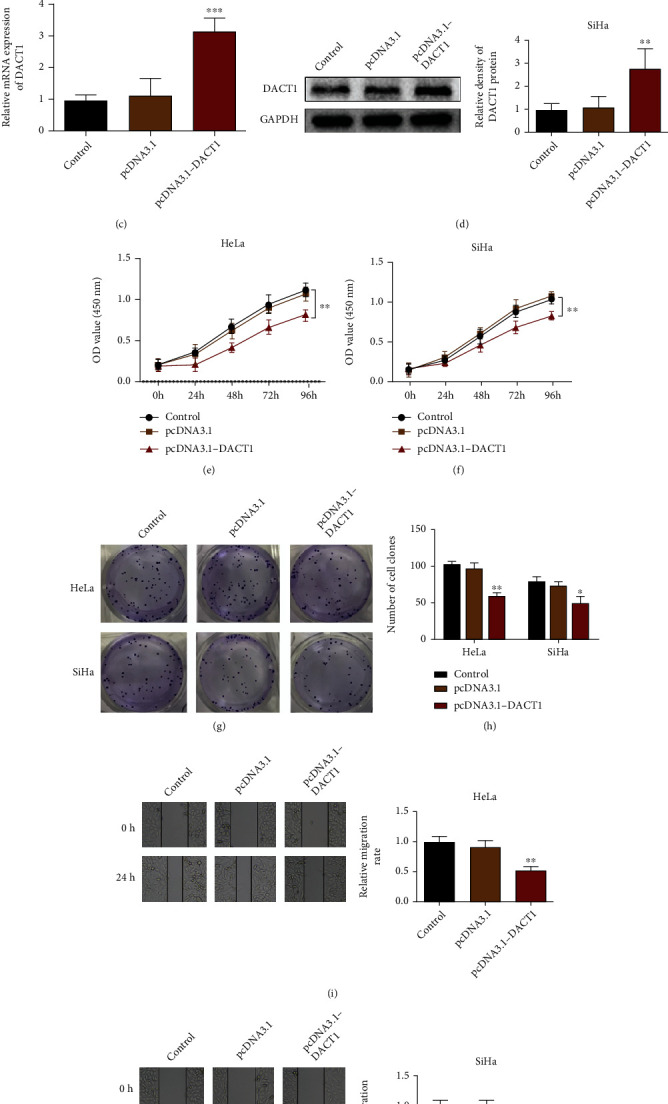
Overexpression of DACT1 leads to suppressed proliferation and migration in both HeLa and SiHa cells. (a) mRNA expression level of DACT1 in HeLa cells after DACT1 overexpression was determined by qRT-PCR; (b) protein expression level of DACT1 in HeLa cells after DACT1 overexpression was determined by western blot; (c) mRNA expression level of DACT1 in SiHa cells after DACT1 overexpression was determined by qRT-PCR; (d) protein expression level of DACT1 in SiHa cells after DACT1 overexpression was determined by western blot; (e) CCK8 assay was applied to assess the effect of DACT1 overexpression on proliferation of HeLa cells; (f) CCK8 assay was applied to assess the effect of DACT1 overexpression on proliferation of SiHa cells; (g, h) cell clone formation assay was used to assess the effect of DACT1 overexpression on cell clones in HeLa cells and SiHa cells; (i, j) wound healing assay analyzed the effect of DACT1 overexpression on cell migration in HeLa and SiHa cells. ^∗^*p* < 0.05, ^∗∗^*p* < 0.01, and ^∗∗∗^*p* < 0.001, compared with the control group.

HeLa and SiHa cells were transfected with DACT1 overexpression through lentiviral transfection. As shown in Figures 3(a)–3(d), the mRNA and protein expressions of DACT1 were substantially increased in HeLa and SiHa cells after pcDNA3.1-DACT1 transfection when compared with the control group (*p* < 0.01). Meanwhile, after pcDNA3.1-DACT1 transfection, the proliferation rate (Figures 3(e) and 3(f), *p* < 0.01), clone formation numbers (Figures 3(g) and 3(h), *p* < 0.01), and migration rate (Figures 3(i) and 3(j), *p* < 0.01) were suppressed in both HeLa and SiHa cell in comparison to those in the control group. The comparison on proliferation rate, clone formation numbers, and migration rate between the pcDNA3.1 group and control group showed no significant difference. Those observations showed that overexpression of DACT1 can inhibit the proliferation and migration of HeLa and SiHa cells.

### 3.4. KDM1A Suppresses DACT1 Expression in Cervical Cancer Cells through Inducing Histone Deacetylation

**Figure 4:**
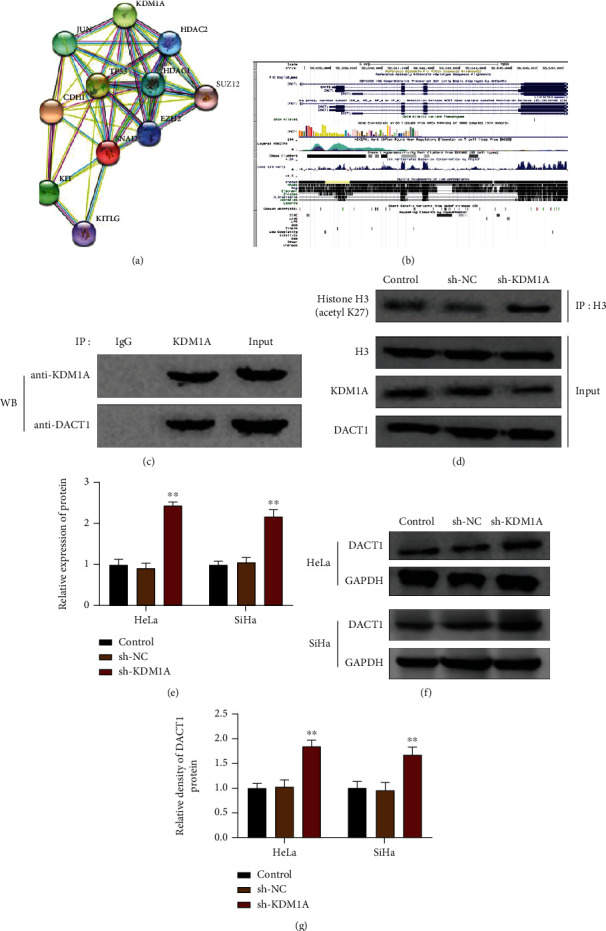
KDM1A negatively regulates DACT1 in cervical cancer cells. (a) STING predicted the interaction between KDM1A and HDAC1; (b) UCSC analysis showed the elevated signals in the prompter of HDAC1 after histone 3 deacetylation; (c) CoIP identified the interaction between KDM1A and DACT1; (d) the deacetylation of histone 3 was measured by western blot; (e) in HeLa and SiHa cells, KDM1A knockdown on mRNA expression level of DACT1; (f, g) in HeLa and SiHa cells, KDM1A knockdown on protein expression level of DACT1. ^∗^*p* < 0.05 and ^∗∗^*p* < 0.01, compared with the control group.

The Genecards database (https://www.genecards.org/) showed that KDM1A can bind with many histone deacetylases to form complex so as to achieve gene silence. The STING database (https://string-db.org/cgi/input.pl?sessionId=4Bvlcg3I4RKk&input_page_show_search=on) predicted the binding between KDM1A and HDAC1 (Figure 4(a)). UCSC analysis demonstrated fluctuations in the DACT1 promoter after histone 3 deacetylation (Figure 4(b)), indicating the DACT1 promoter may be affected by histone 3 deacetylation. Co-IP verified the interaction between KDM1A and HDAC1 (Figure 4(c), *p* < 0.01). Additionally, western blot showed increased deacetylation of histone 3 in sthe h-KDM1A group when compared with the control group (Figure 4(d), *p* < 0.01). The effect of KDM1A knockdown on DACT1 expression in HeLa and SiHa cells showed that compared with the sh-NC group, the sh-KDM1A group had increased mRNA and protein expressions of DACT1 in both HeLa and SiHa cells (Figures 4(e)–4(g), *p* < 0.01), indicating the regulatory role of KDM1A on DACT1 expressions. The collected evidence showed that KDM1A enhances histone 3 deacetylation and therefore suppresses DACT1 expressions.

### 3.5. Overexpression of DACT1 Reverses the Effect of KDM1A Overexpression in Cervical Cancer Cells

**Figure 5:**
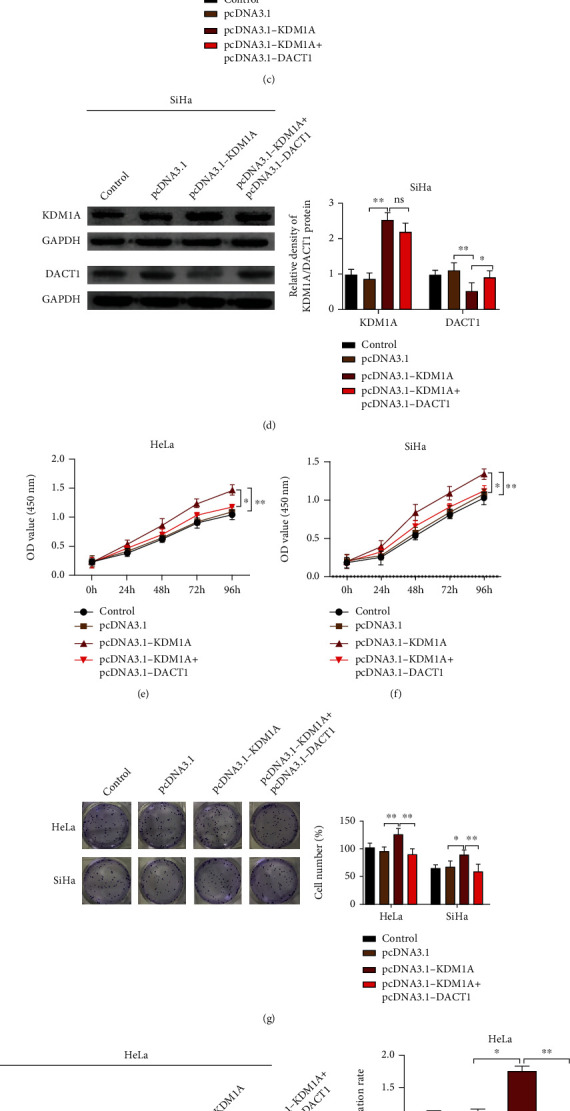
Overexpression of DACT1 reverses the promotive effect of KDM1A overexpression on cervical cancer cells. (a) mRNA expression levels of KDM1A and DACT1in HeLa cells by qRT-PCR; (b) protein expression levels of KDM1A and DACT1in HeLa cells by western blot; (c) mRNA expression levels of KDM1A and DACT1in SiHa cells by qRT-PCR; (d) protein expression levels of KDM1A and DACT1in SiHa cells by western blot; (e) CCK8 assay to measure the cell proliferation ability in HeLa cells; (f) CCK8 assay to measure the cell proliferation ability in SiHa cells; (g) cell clone formation for cell clone count in both HeLa and SiHa cells; (h, i) wound healing assay to measure the migration ability of HeLa cells; (j, k) wound healing assay to measure the migration ability of SiHa cells; ^∗^*p* < 0.05 and ^∗∗^*p* < 0.01. ns: not significant.

To ascertain the effect of DACT1 in cervical cancer, pcDNA3.1-DACT1 and pcDNA3.1-KDM1A were cotransfected into HeLa and SiHa cells. The measurement on transfection efficiency showed that compared with the pcDNA3.1 group, the pcDNA3.1-KDM1A group had higher expression of KDM1A and lower expression of DACT1. Meanwhile, compared with the pcDNA3.1-KDM1A group, the expression of KDM1A showed no significant changes, while DACT1 expression was substantially elevated in the pcDNA3.1-KDM1A+pcDNA3.1-DACT1 group (Figures 5(a) and 5(b), *p* < 0.01).

Measurement on cell proliferation, cell clones, and migration showed that compared with the pcDNA3.1 group, the pcDNA3.1-KDM1A group had increased cell proliferation rate (Figures 5(c) and 5(d), *p* < 0.01) and elevated cell clones (Figures 5(e) and 5(f), *p* < 0.01). In comparison to the pcDNA3.1-KDM1A group, the pcDNA3.1-KDM1A+pcDNA3.1-DACT1 group had suppressed cell proliferation rate (Figures 5(c) and 5(d), *p* < 0.01) and less cell clone (Figures 5(e) and 5(f), *p* < 0.01). Cell migration ability by wound healing assay showed that increased cell migration ability in the pcDNA3.1-KDM1A group when compared with the pcDNA3.1 group, but the pcDNA3.1-KDM1A+pcDNA3.1-DACT1 group had suppressed cell migration ability in comparison with the pcDNA3.1-KDM1A group (Figures 5(g) and 5(h), *p* < 0.01). No significant difference was found between the pcDNA3.1 group and control group in terms of cell proliferation, cell clones, and migration ability. Taken together, KDM1A enhances the proliferation and migration of cervical cancer cells through regulating the expression of DACT1.

## 4. Discussion

In this study, we demonstrated the increased expression level of KDM1A and decreased expression level of DACT1 in both cervical cancer tissues (from TCGA database) and cervical cell lines. More importantly, we further explored the mechanism of KDM1A regulation on DACT1 in cervical cancer. The collected evidence showed that KDM1A in cervical cancer cells can suppress the expression of DACT1 through histone 3 deacetylation and therefore enhance the progression of this disease.

KDM1A is a well-known histone demethylase and an emerging option for the therapeutic treatment of various cancers [15]. KDM1A is typically reported for its overexpression in various kinds of solid tumors and leukemia [16, 17]. For instance, KDM1A can bind CoREST or nucleosome remodeling and deacetylase repressive complex to repress gene transcription [18]. On parallel, KDM1A can also enhance transcriptional activation through interacting with androgen receptor (AR) or estrogen receptor (ER) [18]. The diversity on function of KDM1 should be ascribed to its complex structure and its interactions with transcription factors, promoters, enhances, and tumor suppressor or activators [9]. Consistently, with a previous literature [13], this study demonstrated KDM1A is highly expressed in both cervical cancer tissues and cell lines, whose suppression contributed greatly to the suppression on cell proliferation and migration of cervical cancer cells. In breast cancer cells, the HDAC inhibitor was applied as a therapeutic approach to attenuate disease progression, which was subjected to regulation of crosstalk between KDM1A and histone deacetylation [19]. The histone methylation changes regulated by KDM1A were achieved by removing the methyl groups from the methylated proteins, including histone H3 [20]. Further exploration on KDM1A regulation on biological function of cervical cancer cells in this study showed that KDM1A can induce histone 3 deacetylation so as to suppress the expression of DACT1.

It is not the first time to identify the implication of DACT1 in cervical cancer. A previous study by Shi et al. showed DACT1 by acting as one component of the H1FX-AS1/miR-324-3p/DACT1 axis was proved to be a novel potential therapeutic target for cervical cancer treatment [13]. In light of the genome-wide chromatin-immunoprecipitation study, which has shown that KDM1A binds to the enhancer and promoter regions of genes [20], in this study, we noticed the promoter of DACT1 was influenced by the histone 3 deacetylation. On parallel, Co-IP verified the interaction between KDM1A and DACT1. Therefore, KDM1A may be able to regulate DACT1 expression through histone 3 deacetylation in cervical cancer cell lines. To further identify the possible interaction between KDM1A and DACT1 in cervical cancer cell lines, we then cotransfected overexpression of KDM1A and DACT1 in cervical cancer cell lines. The measurement on cell proliferation and migration ability showed that overexpression of DACT1 could suppress cell proliferation and migration of cervical cancer cells, while overexpression of KDM1A could abolish DACT1-mediated suppression on cervical cancer cells. The tumor suppressive role of DACT1 can be also found in other malignant tumors, including type I ovarian cancer [21], leukemia cells [22], and esophageal squamous cell carcinoma [23]. Evidence in a previous study supported that the epigenetic regulation on DACT1 can lead to expression alternation. Evidence in nasopharyngeal carcinoma showed that DACT1 expression levels in patients with nasopharyngeal carcinoma were closely related to the methylation condition and unregulated expression of DACT1 may be able to suppress the malignant expansion of nasopharyngeal carcinoma cells [24]. Similar with a previous study, the results in this study demonstrated that overexpression of DACT1 can attenuate the progression of cervical cancer, highlighting the tumor suppressing role of DACT1 in cervical cancer. The novelty of this study was KDM1A-mediated histone 3 deacetylation on regulation of DACT1 in cervical cancer. Although this study identified a possible therapeutic approach for cervical cancer treatment, there are several limitations that should be borne in mind. This study mainly focused on the in vitro study, and therefore, the results can be more creditable once the in vivo studies are supplemented, which is one of the future directions for our study. Meanwhile, more validations on our results could be beneficial before targets of KDM1A/DACT1 can be used for clinical trials.

In summary, results in this study showed that KDM1A was highly expressed while DACT1 was lowly expressed in cervical cancer tissues and cells. KDM1A can lead to histone 3 deacetylation, which consequently represses the expression of DACT1 in cervical cancer cells. Additionally, KDM1A is able to negatively regulate the expression of DACT1. Therefore, KDM1A enhances the progression of cervical cancer by inducing histone 3 deacetylation and downregulating DACT1 expression.

## Figures and Tables

**Table 1:** Primer sequences for reverse transcription polymerase chain reaction.

Name of primer	Sequences
KDM1A-F	CGGAATTCGGCGGCCCGAGATGTTAT
KDM1A-R	CCCTCGAGTGGGCCTCTTCCCTTAGAAT
DACT1-F	GACAGACAGTCGGCCTAGCTCA
DACT1-R	AGAGACTCAAGGTCGCCTCCAA
GAPDH-F	GTCGATGGCTAGTCGTAGCATCGAT
GAPDH-R	TGCTAGCTGGCATGCCCGATCGATC

F: forward; R: reverse.

## Data Availability

All data are accessible upon reasonable request to the corresponding author.
